# Does stereoscopic imaging improve the memorization of medical imaging by neurosurgeons? Experience of a single institution

**DOI:** 10.1007/s10143-021-01623-0

**Published:** 2021-09-22

**Authors:** Nicolas Schlinkmann, Rutvik Khakhar, Thomas Picht, Sophie K. Piper, Lucius S. Fekonja, Peter Vajkoczy, Gueliz Acker

**Affiliations:** 1 Charité – Universitätsmedizin Berlin, corporate member of Freie Universität Berlin and Humboldt-Universität zu Berlin, Department of Neurosurgery, Charitéplatz 1, 10117 Berlin, Germany; 2 Charité – Universitätsmedizin Berlin, corporate member of Freie Universität Berlin and Humboldt- Universität zu Berlin, Cluster of Excellence: “Matters of Activity. Image Space Material”, Charitéplatz 1, 10117 Berlin, Germany; 3 Charité – Universitätsmedizin Berlin, corporate member of Freie Universität Berlin and Humboldt-Universität zu Berlin, Institute of Biometry and Clinical Epidemiology, Charitéplatz 1, 10117 Berlin, Germany; 4 Berlin Institute of Health at Charité – Universitätsmedizin Berlin, BIH Academy, Clinician Scientist Program, Charitéplatz 1, 10117 Berlin, Germany

**Keywords:** 3D, Stereoscopic, Memorization, Imaging, Neurosurgery

## Abstract

Stereoscopic imaging has increasingly been used in anatomical teaching and neurosurgery. The aim of our study was to analyze the potential utility of stereoscopic imaging as a tool for memorizing neurosurgical patient cases compared to conventional monoscopic visualization. A total of 16 residents and 6 consultants from the Department of Neurosurgery at Charité – Universitätsmedizin Berlin were recruited for the study. They were divided into two equally experienced groups. A comparative analysis of both imaging modalities was conducted in which four different cases were assessed by the participants. Following the image assessment, two questionnaires, one analyzing the subjective judgment using the 5-point Likert Scale and the other assessing the memorization and anatomical accuracy, were completed by all participants. Both groups had the same median year of experience (5) and stereoacuity (≤ 75 s of arc). The analysis of the first questionnaire demonstrated significant subjective superiority of the monoscopic imaging in evaluation of the pathology (median: monoscopic: 4; stereoscopic: 3; *p* = 0.020) and in handling of the system (median: monoscopic: 5; stereoscopic: 2; *p* < 0.001). The second questionnaire showed that the anatomical characterization of the pathologies was comparable between both visualization methods. Most participants rated the stereoscopic visualization as worse compared to the monoscopic visualization, probably due to a lack of familiarity with the newer technique. Stereoscopic imaging, however, was not objectively inferior to traditional monoscopic imaging for anatomical comprehension. Further methodological developments and incorporation in routine clinical workflows will most likely enhance the usability and acceptance of stereoscopic visualization.

**Supplementary Information:**

The online version contains supplementary material available at 10.1007/s10143-021-01623-0.

## Introduction

In medicine, stereoscopic (stereo) screens have been increasingly utilized in teaching, preoperative planning, and also during surgery [1–5]. Due to depth perception created by the projection of a slightly different image to each eye, stereoscopic displays allow a more accurate visualization compared to three-dimensional (3D) images on two-dimensional (2D) displays [6]. In this regard, Harake et al. demonstrated that interactive stereoscopic visualization of three-dimensional echocardiography was preferred over conventional display by cardiologists, advanced cardiac trainees, and surgeons in viewing both simple and complex congenital cardiac lesions [7]. Surgical disciplines, like neurosurgery, orthopedics, or visceral surgery, are particularly interested in new ways to visualize complex anatomical relations during surgical planning [1, 4, 5]. However, the benefits of stereoscopic imaging for clinical routine are still controversial. While in a recent study we were able to show an advantage of stereoscopic imaging in the detection of challenging aneurysms, Stewart et al. could not detect any benefits with stereoscopic viewing of volume-rendered three-dimensional computed tomography angiograms (CT-A) in the characterization of cerebral aneurysms compared with monoscopic (mono) viewing [8, 9].

Memorization of the individual anatomy is particularly important for surgeons. For medical students, it has already been shown that hand-made drawings can be helpful in memorizing anatomical structures [10]. Furthermore, three-dimensional programs have already proven to be advantageous in preoperative planning and in anatomical teaching [11, 12]. However, Park et al. demonstrated that a three-dimensional anatomical atlas for first-year medical students could not enhance the memorization of anatomical structures [13]. Importantly, a recent meta-analysis by Bogomolova et al. has shown that for learning anatomy, stereoscopic 3D is superior to monoscopic 3D [14]. The additional advantage of stereoscopic visualization compared to viewing 3D images on 2D displays, which is more prevalent in routine clinical practice, was highlighted in this report [14]. In terms of adoption, a high level of acceptance for stereoscopic 3D videos by students has been validated with an added benefit in anatomical understanding in a very recent study [15].

With regard to preoperative planning, the effectiveness of 3D visualization for neurosurgical interventions was reported back in 1996 [16]. Preoperative planning with interactive 3D computed tomography (CT) reconstruction has also been proven to be a useful method to enhance the surgeon’s knowledge of the patient’s individual anatomy in thoracoscopic lung surgery [17]. Similarly, a neurosurgeon’s efficiency could be greatly improved by better understanding and memorizing the neuroanatomy prior to surgery using stereoscopic imaging. The aim of the present study is, therefore, to investigate, first on one hand, the subjective advantage of stereoscopic imaging for preoperative planning and, on the other hand, to evaluate the effect of stereoscopy on the memorization of anatomical information in patient cases of neurosurgery.

## Methods

### Study design

The study was performed in accordance with the ethical standards of the Declaration of Helsinki. The ethics committee of Charité – Universitätsmedizin Berlin approved the present retrospective analysis of data that was collected during department teaching (approval number, EA1/090/20). The imaging studies from patients treated at our department for subarachnoid hemorrhage from 2009 to 2018 and for brain tumor with preoperative fiber tracking from 2018 were reviewed to choose suitable cases for the teaching sessions. Two representative tumor and vascular surgical cases were selected based on predefined criteria, namely a tumor with involvement of the motor fiber tract and one with an affected language fiber tract, as well as an aneurysm in the anterior circulation and one in the posterior circulation.

**Fig. 1:**
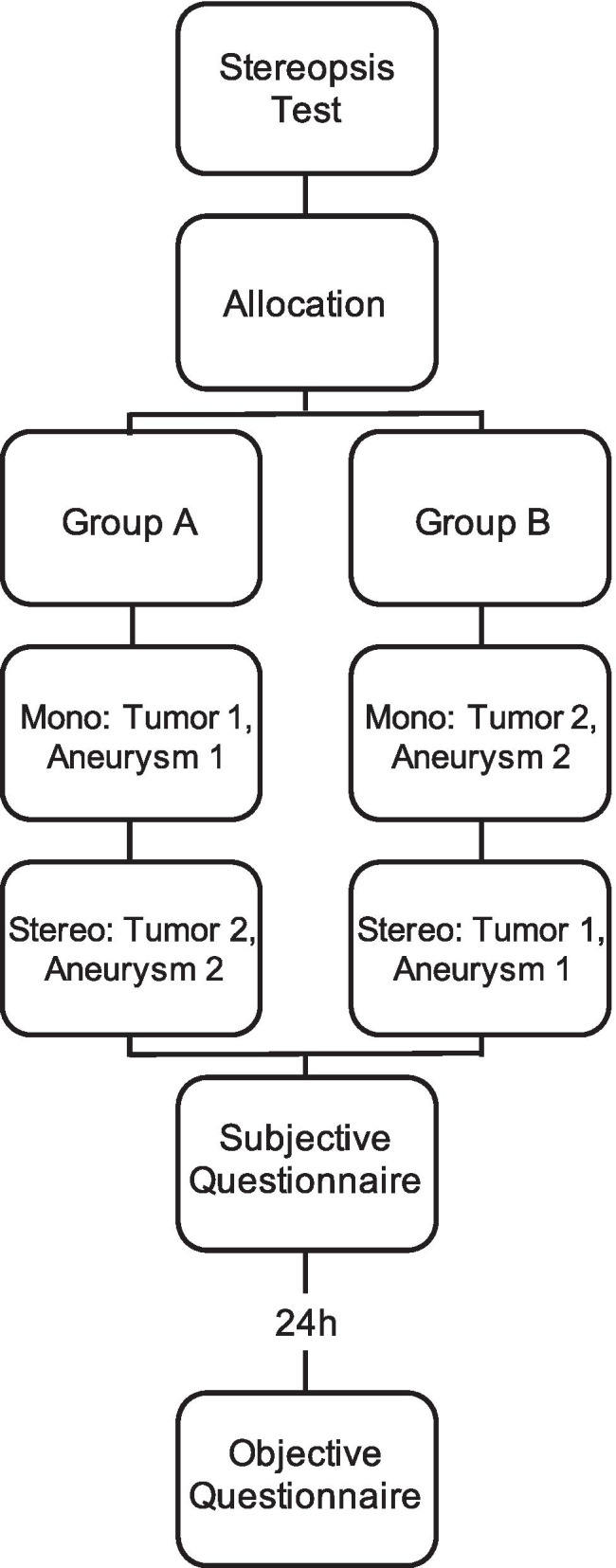
Study design

Neurosurgeons in our department who attended this teaching session were included in the study. Participants were assigned to two balanced groups based on their experience level and stereopsis was tested with the Frisby Pocket Stereotest™. Group A first assessed a frontal brain tumor and a middle cerebral artery bifurcation aneurysm, while group B first assessed a central brain tumor and a vertebral artery aneurysm using the monoscopic visualization system. Shortly thereafter, the groups switched and assessed the cases of the other group using the stereoscopic visualization system (Fig. 1). All participants were briefly introduced to the handling of the stereoscopic visualization system before analyzing the cases with it. Each participant was given a maximum of 5 min per case to evaluate the images in each block for themselves. The time taken to identify the aneurysm during the image analysis was recorded. It is important to note that those participants who failed to detect an aneurysm were not included in the assessment of detection time. However, regarding the time to analyze the pathologies, all the participants for both visualization systems were included. In cases where the aneurysm could not be detected by the participant, the duration of the searching process (max. 5 min) was taken instead.

After analyzing the images with both visualization systems, all participants completed a subjective feedback questionnaire. The questionnaire obtained parameters such as the ability to recognize pathological and anatomical structures, amount of radiological information perceived, preferred visualization system, and confidence in using the visualization systems (supplemental Fig. 1). Participants were not informed about the content of the following day’s re-evaluation but were notified that questions relating to the first day’s procedure would be asked.

The following day, participants were asked to complete a questionnaire on the specifications of the pathology, such as the exact location, morphology, and invasion of associated tracts to assess the objective gain and retention of the information.

(supplemental Fig. 2−3).

### Characteristics of the selected surgical cases

**Fig. 2:**
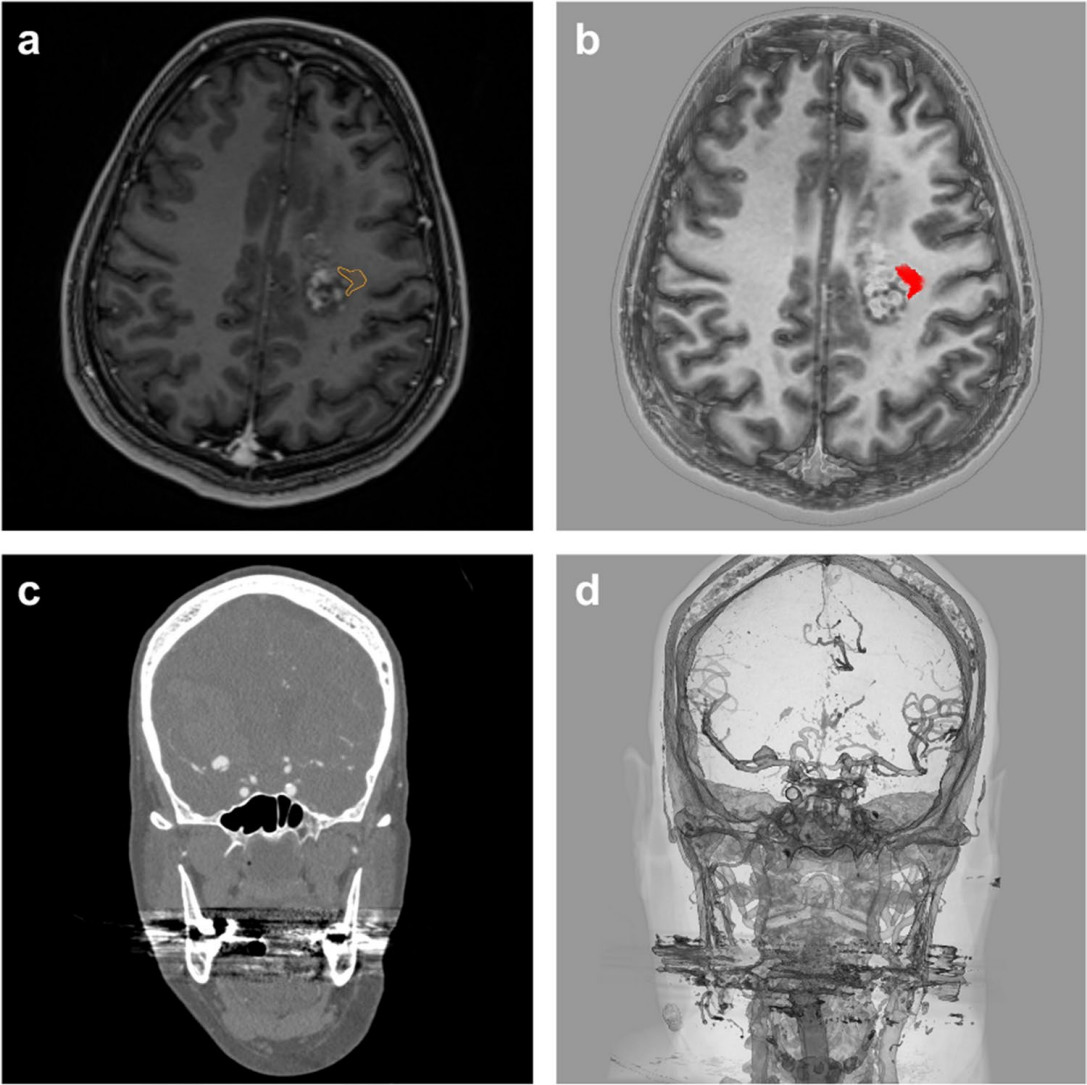
In the top row (**a** + **b**), an axial view of the T1-weighted magnetic resonance imaging of the central tumor (tumor 1), monoscopically with Brainlab (**a**) and stereoscopically with VP Reveal (**b**). The tract is shown in orange for Brainlab and red for VP Reveal. In the lower row (**c** + **d**), a coronal view of the computed tomography angiography of a middle cerebral artery bifurcation aneurysm (aneurysm 2), monoscopically with Brainlab (**c**) and stereoscopically with VP Reveal (**d**)

The tumor pathologies were two left-sided recurrent glioblastoma, one central with an invasion of the corticospinal tract (tumor 1; size: 13 × 25 × 20 mm) and one in the middle frontal gyrus that extended to the frontal operculum involving the uncinate fascicle (tumor 2; size: 33 × 18 × 31 mm) (Fig. 2a–b). A right-sided vertebral artery fusiform aneurysm (aneurysm 1; 5 × 7 mm) and a right-sided middle cerebral artery saccular aneurysm (aneurysm 2; 9 × 7 mm) were selected for the neurovascular cases (Fig. 2c–d).

### Image processing and viewing

Using the program VPI Reveal, version 1.5 (Vesalius Perfectus International BV, Eindhoven, the Netherlands), 3D volume rendering and display of the magnetic resonance imaging (MRI) and CT-A Digital Imaging and Communications in Medicine (DICOM) images were performed. VPI Reveal is a proprietary software based on inhouse developed 3D volume rendering technology using Raytracing technology and dedicated image post processing to facilitate optimal stereoscopic 3D display (Holographic 3D). The DICOM images were imported from the hospital’s picture archiving and communication system (PACS) into the VP Reveal software, using the integrated import system, and the software automatically generated a 3D model. Subsequently, we could modify the visualization settings to improve the quality. For this purpose, each Hounsfield unit could be assigned with a specific gray value or a color. For the tumor and vascular system, an individual grayscale was chosen, while the imported fiber tracts were colored red. In each case, contrast and transparency of the images were adjusted so that an individual 3D model could be generated. This process required approximately 3 min per case. For image navigation, a 3D mouse (3Dconnexion SpaceMouse Compact) was used. The study was conducted at two different hospital sites, using a high-resolution liquid crystal display (LCD) monitor (Hyundai S465D; 3D LCD monitor, 46 in.; resolution: 1920 × 1080; 60 Hz) and a high-resolution light-emitting diode (LED) monitor (LG 55LA6208; 3D LED monitor, 55 in.; resolution: 1920 × 1080; 200 Hz MCI) for 3D display, respectively. Commercially available polarized 3D glasses by Hyundai and LG were used. The VP Reveal was running on a Dell inc. Precision 7720 computer with an Intel Core™ i7-7920HQ (3.10 GHz) central processing unit and an NVIDIA Quadro P4000 graphics card. To evaluate the monoscopic (multiplanar reformation processed) MRI and CT-A images, a FUJITSU ESPRIMO D956 computer with an Intel Core™ i5-6500 (3.20 GHz) central processing unit and an Intel HD Graphics 530 graphics card were used at both sites. The DICOM images were viewed via iPlan Net 3.0 (Brainlab, Munich) using a computer display monitor (Fujitsu B22T-7, 21.5 in.; resolution: 1920 × 1080).

### Statistical analysis

Descriptive statistics were given as median with limits of the interquartile range (IQR) [25th–75th percentile]. Wilcoxon’s signed-rank test was used to compare each participant’s subjective perception of monoscopic viewing with the corresponding stereoscopic viewing assessment, independent of group A or B. Objective perception was assessed as the number of correct responses per case for each participant in the second questionnaire. The total point of correct answers was 8 for each tumor and 7 for each aneurysm resulting from 8 and 7 different questions, respectively. The Mann–Whitney *U* test was used to compare the objective perception rates between viewing modalities (monoscopic vs. stereoscopic) for each surgical case (tumor 1, tumor 2, aneurysm 1, aneurysm 2). The years of experience were correlated with the objective perception for each viewing modalities (monoscopic vs. stereoscopic) using the Spearman rank correlation coefficient. All tests were 2-sided, and *p* < 0.05 was considered to indicate statistical significance. All tests should be understood as constituting an exploratory analysis and no adjustment for multiple testing was made. The statistical analyses were performed using SPSS, version 25 (International Business Machines Corporation (IBM), Armonk, NY). Graphs were made using GraphPad Prism, version 8 (GraphPad Software, San Diego, CA) and edited using PowerPoint, version 16 (Microsoft, Redmond, WA). Data and tables were managed with Excel, version 16 (Microsoft, Redmond, WA).

## Results

### Characterization of the participants

**Fig. 3:**
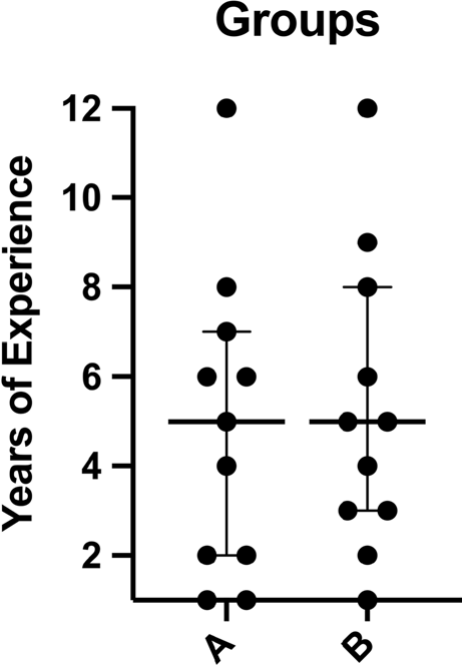
Comparison of both groups for years of experience with 11 participants in each group. The dots represent each participant’s year of experience; the lines represent the median and the interquartile range (25th–75th percentile). Group A: years of experience: median 5, IQR 2–7. Group B: years of experience: median 5, IQR 3–8

A total of 22 neurosurgeons participated as raters with different experience level (range: 1–12 years of experience), half of them (*n* = 11) were allocated into group A and the other half into group B. In each group, the median year of experience was 5 (Fig. 3). There were 10 female (45.5%) and 12 (54.5%) male participants. All participants had stereopsis with the same median of 75 s of arc (s arc) (Group A: median 75 s arc, IQR 75–90 s arc. Group B: median 75 s arc, IQR 75–75 s arc).

### Subjective analysis

**Fig. 4:**
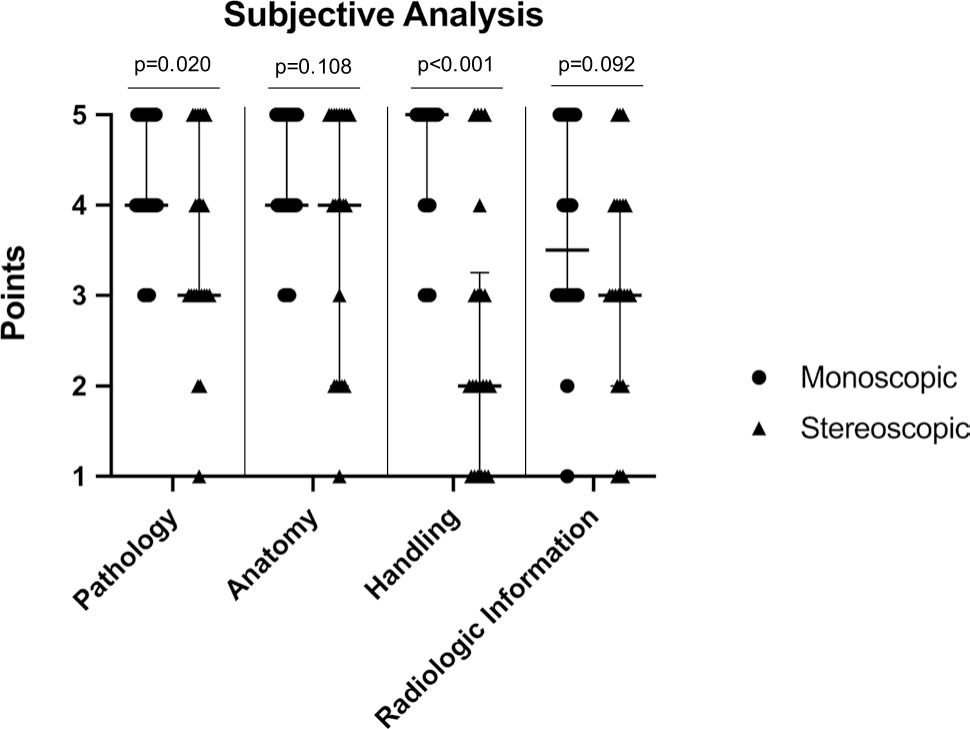
Comparison of the subjective analysis of both image viewing modalities with 22 participants (Wilcoxon signed-rank test). The dots and triangles represent the points awarded by each participant for the various categories. Additionally, the lines represent the median and the interquartile range (25th–75th percentile). Pathology: mono: median 4, IQR 4–5; stereo: median 3, IQR 3–5. Anatomy: mono: median 4, IQR 4–5; stereo: median 4, IQR 2–5. Handling: mono: median 5, IQR 4–5; stereo: median 2, IQR 1–3.25. Radiologic information: mono: median 3.5, IQR 3–5; stereo: median 3, IQR 2–4

The questionnaire at the end of the first day, that documented the participants’ subjective perception, revealed a significant advantage of the monoscopic imaging in pathology assessment and subjective device handling (Fig. 4). The identification of the anatomy and the processing of the radiologic information did not differ between both modalities (Fig. 4). Overall, more than half of the participants (*n* = 14, 63.6%) preferred the monoscopic imaging over stereoscopic, six (27.3%) favored stereoscopy, while two (9.1%) had no preference.

When comparing residents and consultants, the two modalities were rated similarly (Table 1). All but one of the medians differed no more than 0.5 points between both groups. The most relevant difference between residents and consultants was in the extraction of the radiologic information (median [IQR] residents: 3 [3–4.25], consultants: 4.5 [3.25–5]; *p* = 0.261; Mann–Whitney *U* test).

**Table 1:** Itemized subjective analysis comparison of residents and consultants

		Pathology		Anatomy		Handling		Radiologic information	
		Mono	Stereo	Mono	Stereo	Mono	Stereo	Mono	Stereo
Residents (*n* = 16)	Median	4	3	4.5	4	4.5	2	3	3
	25th percentile	4	3	4	3.5	3.75	1	3	2.75
	75th percentile	5	5	5	5	5	3.25	4.25	4
Consultants (*n* = 6)	Median	4	3.5	4	3.5	5	2.5	4.5	3.5
	25th percentile	4	3	4	2.25	5	2	3.25	2.25
	75th percentile	4.75	4	4.75	4.75	5	3	5	4.75

### Objective analysis

**Fig. 5:**
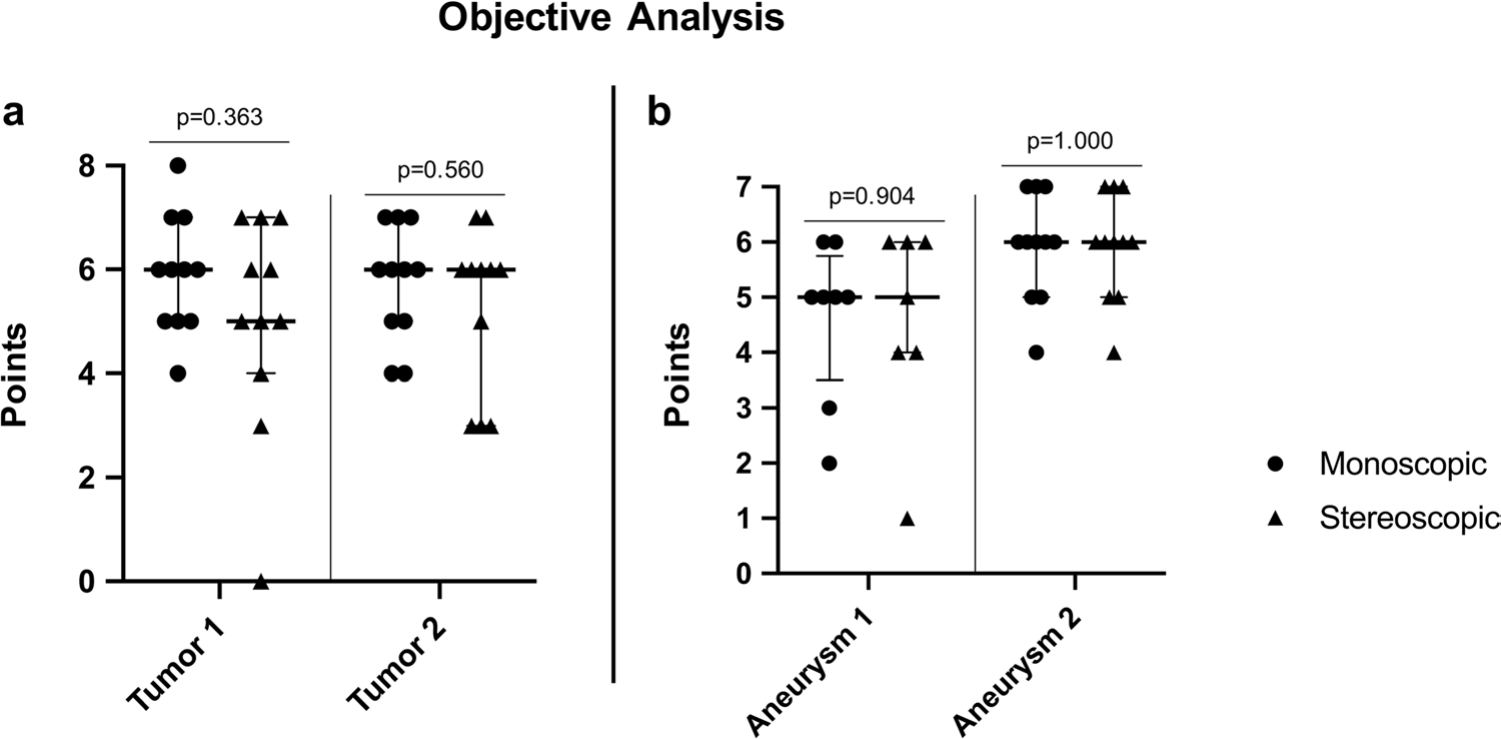
**a** Comparison of the achieved points in evaluating the tumor patients for monoscopic and stereoscopic imaging systems with 11 participants for each tumor (Mann–Whitney *U* test). The dots and triangles represent the achieved points by each participant for both tumors; the lines represent the median and the interquartile range (25th–75th percentile). **b** Comparison of the achieved points in evaluating the aneurysm patients for monoscopic and stereoscopic imaging systems with 8 (monoscopic) and 7 (stereoscopic) participants for aneurysm 1 and 11 participants in each group for aneurysm 2 (Mann–Whitney *U* test). The dots and triangles represent the achieved points by each participant for both aneurysms; the lines represent the median and the interquartile range (25th–75th percentile)

In the second questionnaire on the following day, the objective gain of information was analyzed based on the points obtained by describing the localization and morphology of the pathologies. The evaluation of the pathologies did not differ significantly in any of the cases (Fig. 5). During the assessment, seven (31.8%) participants could not detect the aneurysm in posterior circulation, three of group A (27.3%, monoscopic) and four of group B (36.4%, stereoscopic).

The comparison between residents and consultants highlighted that the various pathologies could be assessed equally well by both experience groups using both modalities without any significant differences in median (Table 2). The largest discrepancy between residents and consultants was in the objective analysis of tumor 2 by monoscopic visualization (median [IQR]: residents: 5.5 [4.75–6], consultants: 7 [6.5–7]; Mann–Whitney *U* test *p* = 0.085).

**Table 2:** Itemized objective analysis comparison of residents and consultants for both tumors (T1 and T2) and aneurysms (A1 and A2)

		T1 mono	T1 stereo	T2 mono	T2 stereo	A1 mono	A1 stereo	A2 mono	A2 stereo
Residents (*n* = 8)*	Median	6	5	5.5	6	5 (*n* = 6)	5 (*n* = 5)	6	6
	25th percentile	5	3.75	4.75	4.5	3.5	4	5.75	5
	75th percentile	7	6.25	6	6	5	6	6.25	6.25
Consultants (*n* = 3)*	Median	6	6	7	6	5.5 (*n* = 2)	5 (*n* = 2)	6	6
	25th percentile	5.5	5.5	6.5	4.5	5.25	4.5	5.5	6
	75th percentile	6	6.5	7	6.5	5.75	5.5	6.5	6.5

^*^Number of participants is stated, if it differs from here

### Duration of the pathology assessment

**Fig. 6:**
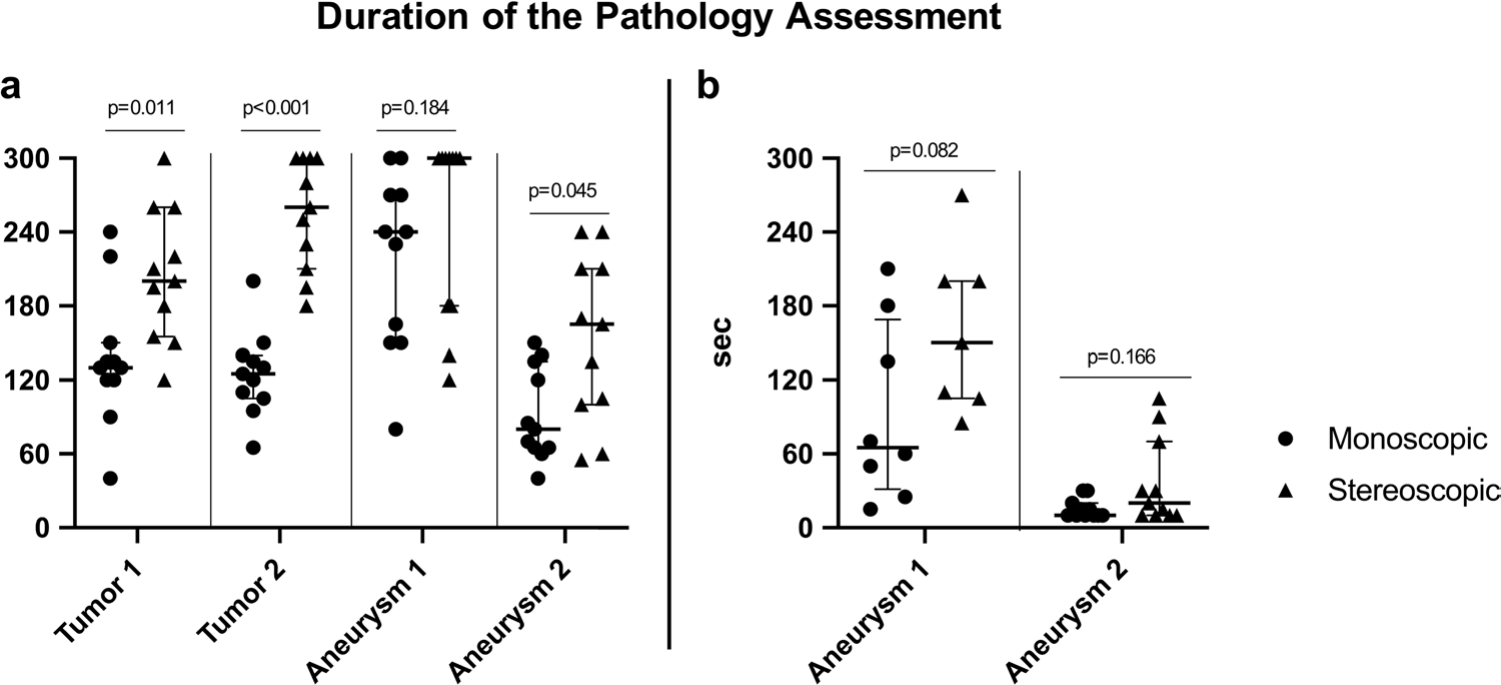
**a** Comparison of the time to evaluate the different pathologies for monoscopic and stereoscopic imaging system with 11 participants for each pathology and imaging system. The time to analyze the pathologies was significantly less using the monoscopic imaging system except in aneurysm 1 (Mann–Whitney *U* test). The dots and triangles represent the time needed by each participant to analyze the different pathologies; the lines represent the median and the interquartile range (25th–75th percentile). **b** Comparison of the time to detect the aneurysm for monoscopic and stereoscopic imaging systems with 8 (monoscopic) and 7 (stereoscopic) participants for aneurysm 1 and 11 participants for aneurysm 2 (Mann–Whitney *U* test). The dots and triangles represent the time needed by each participant to find the aneurysm; the lines represent the median and the interquartile range (25th–75th percentile)

The time to analyze the pathologies was significantly longer using the stereoscopic imaging system for tumor 1, tumor 2, and aneurysm 2 (Fig. 6a), while it was comparable for the aneurysm 1 in the posterior circulation (Fig. 6a). For both imaging modalities, the time to detect the aneurysms was in the same range (Fig. 6b).

When comparing residents and consultants, the most obvious difference was observed for the aneurysm detection in posterior circulation (aneurysm 1) using the stereoscopic imaging system: The consultants required 210 s in median [IQR: 180–240] to find the aneurysm, while the residents required only 110 s in median [IQR: 105–200] (Table 3). For the overall time needed for the analysis of aneurysm 1 using the monoscopic imaging system, the residents required more time (Table 4) (median [IQR]: residents: 255 [237.5–277.5] s, consultants: 150 [115–157.5] s).

**Table 3:** Itemized time todetect the aneurysms (in seconds)with comparison of residents and consultants

		A1 mono	A1 stereo	A2 mono	A2 stereo
Residents	Median	102.5 (*n* = 6)	110 (*n* = 5)	15 (*n* = 8)	15 (*n* = 8)
	25th percentile	62.5	105	10	10
	75th percentile	168.75	200	22.5	30
Consultants	Median	37.5 (*n* = 2)	210 (*n* = 2)	10 (*n* = 3)	70 (*n* = 3)
	25th percentile	31.25	180	10	42.5
	75th percentile	43.75	240	10	87.5

**Table 4:** Itemized time to analyze the pathologies (in seconds) with comparison of residents and consultants

		T1 mono	T1 stereo	T2 mono	T2 stereo	A1 mono	A1 stereo	A2 mono	A2 stereo
Residents (*n* = 8)	Median	132.5	205	122.5	265	255	300	82.5	152.5
	25th percentile	112.5	191.25	106.25	206.25	237.5	180	68.75	90
	75th percentile	167.5	230	138.75	300	277.5	300	136.25	217.5
Consultants (*n* = 3)	Median	130	150	130	260	150	300	65	165
	25th percentile	125	135	117.5	245	115	220	62.5	135
	75th percentile	132.5	205	135	280	157.5	300	92.5	187.5

### Correlation analysis

**Fig. 7:**
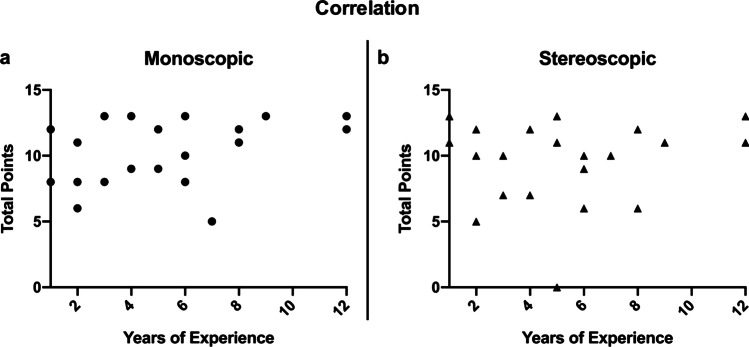
**a** Correlation of the years of experience and the total points achieved using the monoscopic visualization system (Spearman rank correlation coefficient; ρ = 0.289; *p* = 0.193). The dots represent each participant’s achieved points and their year of experience. **b** Correlation of the years of experience and the total points achieved using the stereoscopic visualization system (Spearman rank correlation coefficient; ρ =  − 0.005; *p* = 0.982). The dots represent each participant’s achieved points and their year of experience

The total points of each participant for both cases per visualization system were added in order to correlate the objective performance in the second questionnaire with the level of experience. The total points that the participants had achieved after using the monoscopic or stereoscopic imaging system were not correlated to their experience level (Fig. 7).

## Discussion

In this study, we analyzed the value of stereoscopic visualization for memorization on the basis of four different neurosurgical cases in a group of 22 neurosurgeons with various experience levels. The results demonstrate that the accuracy of stereoscopic assessment of neurosurgical pathologies was comparable to the monoscopic visualization used routinely. However, the subjective perception ranked the stereoscopic imaging worse, most likely due to the lack of prior experience with 3D imaging in general and the used system in particular.

The use of three-dimensional non-stereoscopic reconstructions has already been investigated in several studies in different fields [17–19]. For instance, students that learned gastrointestinal anatomy with 3D reconstructions achieved superior test results and were more skillful at dissection [19]. Glittenberg et al. demonstrated that 3D animations improved the understanding of ophthalmic anatomy and physiology [18]. For neurosurgery, Kikinis et al. reported the benefit of three-dimensional visualization to gain otherwise almost inaccessible information about the spatial relations that could be used for the surgical removal of brain lesions [16]. A systematic review by Arantes et al. concluded that 3D models were an effective way to teach neuroanatomy [20]. Despite these studies demonstrating the advantages of 3D visualization in teaching and preoperative planning, Park et al. could not detect a benefit of the three-dimensional anatomical atlas for the memorization of anatomical structures for first-year medical students [13]. Stereoscopy on the other hand offers a better depth sensation of the 3D images. Additionally, according to the cognitive load theory, more mental connections are activated during stereoscopic viewing that could possibly support the memorization process [21]. Stereoscopic imaging systems have also proven to demonstrate a significant improvement in the acquisition of minimally invasive surgical skills for novice surgeons as compared to the standard 2D imaging system [22–24]. For instance, in a study by Schoenthaler et al. utilizing a surgical simulator model for laparoscopic surgery, stereoscopic imaging led to an improvement while performing the laparoscopic phantom tasks [22]. Likewise, advantages of stereoscopic three-dimensional imaging in teaching anatomy have already been identified in the past [15]. Stadie et al. were able to show that through virtual reality planning the spatial understanding of the anatomy of neurosurgical cases could be improved in 55.8% of the participants and only 2.4% reported no improvement [25]. Brown et al. have also demonstrated that subjectively students achieved a better understanding of anatomy and pathology through stereoscopic three-dimensional images [26]. Furthermore, a recently published meta-analysis focusing on the anatomy learning supported these findings and highlighted the advantage of stereoscopic 3D over non-stereoscopic 3D [14]. However, the potential of three-dimensional volume rendering for stereoscopic visualization has not been fully exploited [27]. Despite these studies in support of stereoscopic visualization in education, Chytas et al. recently discussed the methodological inadequacy of some of the previously published comparative studies and encouraged further investigations [28].

An important aspect of evaluating the usefulness of stereoscopy is one’s experience in the field and the area of ​​application being examined. In both above-mentioned studies, the advantages of the stereoscopy in teaching were mainly seen amongst students who had no professional experience in clinical routine [15, 26]. On one hand, since the younger generation is exposed to three-dimensional visualization much earlier, they can naturally feel more familiar and confident with this type of imaging. On the other hand, the advantage of stereoscopy in diagnostics, for example, could wane with increasing professional experience in the application of monoscopic visualizations, which enables a better understanding of 2D imaging and the ability to reconstruct images in the head. Consistent with this assumption, we could only find a benefit in detecting aneurysms using stereoscopic visualization for residents and not for consultants in our previous study [8]. However, due mainly to the limited number of participants, we were unable to detect any correlation between the years of experience and the points achieved in the present study. This was also supported by the lack of significant differences in individual categories between the residents and consultants. In this context, however, we would like to point out that stereoscopy has a wide range of applications in medicine that is not limited to diagnostics or teaching. During an operation in particular, the sense of depth is of great importance when using a surgical microscope, in which stereoscopy certainly retains its advantages regardless of experience level.

Importantly, both assessment groups (A and B) in our study were overall comparable regarding neurosurgical experience and stereoacuity. The capacity to perceive stereoscopic images is the primary requirement for stereoscopy. According to Odell et al., a stereoacuity of > 250 s arc is considered insufficient [29]. All of our participants had sufficient stereopsis, that highlights the possible routine application of this technique in daily clinical life. This can be supported by the results of other studies for surgeons in which only 2–14% (depending on the test) of the participating surgeons had impaired stereopsis [30]. Furthermore, Chopin et al. estimated the prevalence of “stereoblindness” in adults under 60 years is only about 7% [31].

One major issue in the implementation of stereoscopic visualization in the clinical routine for image assessments, nevertheless, is the prerequisite training required to familiarize the user with this or similar specific systems. Importantly, neurosurgeons are usually familiar with deep immersion through stereoscopic visualization, as it is routinely used in the surgical microscope. Adaptation to stereoscopic viewing is therefore not a major problem. However, different handling is required for the evaluation of the MRI or CT imaging with stereoscopic visualization. In the subjective analysis of our participants, the stereoscopic visualization seemed to have no advantages over the conventional imaging technique. In fact, using the new system turned out to be more complicated and less intuitive for the majority, especially in handling. This could be partly explained by a lack of prior training with this specific system, particularly with regard to scrolling with the 3D mouse. It is also of interest that participants rated the stereoscopic presentation of aneurysm cases better than that of the tumor cases. A similar result was also shown by Bairamian et al., who observed an advantage of stereoscopy in the visualization of challenging cerebrovascular anatomy [32]. This might be due to the better visualization of the vessels by colored contrast adjustments. Overall, the data so far indicate a benefit of stereoscopic visualization in the diagnostic way for selected cases, so this new technology should be implemented as a supplement to conventional 2D visualization.

It is of major importance that the objective results did not coincide with the subjective assessment that the pathologies were worse to assess. In the objective analysis, the stereoscopic visualization was not inferior to the monoscopic one despite lack of prior training with the new system. The individual pathologies were assessed comparable by both groups using both systems. As the pathologies themselves were not easily comparable, an analysis between the two systems per participant as performed in our previous study was omitted here [8]. For instance, in contrast to the second aneurysm, the first aneurysm could not be detected by all participants. Contrary to our previous results, more participants during the stereoscopic visualization system session were unable to find the aneurysm in the posterior circulation [8]. This could be because the participants in this study had to scroll through the images themselves. The video in the Appendix illustrates an appropriate overview of the aneurysms (video 1).

The lack of familiarity with the stereoscopic system could also explain the longer time required to analyze the cases. A longer processing time was also revealed by the results of the examination of 3D echocardiography [7]. Even so, the aneurysms were diagnosed equally quickly on both imaging systems and the residents were able to identify both aneurysm cases faster using the stereoscopic system compared to the consultants. This may be due to the variation in exposure of different generational cohorts to such technologies. On the other hand, Itatani et al. stated that stereoscopic visualization could reduce the operative time in laparoscopic gastrectomy when used by trained surgeons [33]. In our study, all expertise levels handled the monoscopic imaging system more quickly, presumably due to its use in the routine clinical practice and a lack of training with the newer technology.

The main limitation of our study is the low number of participants and cases presented due to its monocentric design. In addition, the lack of prior training with this specific stereoscopic system may have diminished the potential benefits of the new imaging modality. In this context, we noticed that even in the stereoscopic session, participants tended to use the conventional levels during image assessments. This could also be one of the reasons that the full potential of stereoscopic imaging may not have been verified. Another potential point of criticism is the comparison between the stereoscopic 3D imaging with only 2D images as discussed by Chytas et al. [28]; however, our goal was to evaluate the added value of stereoscopy to the 2D images in clinical routine.

## Conclusion

Monoscopic visualization was preferred by most study participants in comparison to the unaccustomed stereoscopic system. Stereoscopic imaging, however, was not inferior to conventional monoscopic imaging for memorization. In the future, more methodological developments and incorporation in routine clinical workflows will most likely increase the usability and acceptance of stereoscopic visualization. Therefore, further, preferably multicenter studies on this topic are warranted.

## Supplementary Information

Below is the link to the electronic supplementary material.

Supplementary file1 (DOCX 28 KB)

Supplementary file2 (PDF 80 KB)

Supplementary file3 (PDF 195 KB)

Supplementary file4 (PDF 98 KB)

Supplementary file5 (DOCX 16 KB)

Supplementary file6 (MOV 10017 KB)

## Data Availability

The datasets generated during and/or analyzed during the current study are available from the corresponding author on reasonable request.
