# Supplementary material for: Does stereoscopic imaging improve the memorization of medical imaging by neurosurgeons? Experience of a single institution
Source: Neurosurg Rev. 2021 Sep 22;45(2):1371–81. doi: 10.1007/s10143-021-01623-0 (PMC8976776; doi:10.1007/s10143-021-01623-0)
Supplement: Supplementary file 1 — Supplementary file1 (DOCX 28 KB) [file 10143_2021_1623_MOESM1_ESM.docx]

| **Supplemental Table 1. Results for questionnaire day 1: subjective comparison of monoscopic vs. stereoscopic imaging method** | | | | | | | | | | |
| --- | --- | --- | --- | --- | --- | --- | --- | --- | --- | --- |
| **Participant** | **Year of experience** | **Pathology** | | **Anatomy** | | **Handling** | | **Radiologic Information** | | **Preferation** |
|  |  | **mono** | **stereo** | **mono** | **stereo** | **mono** | **stereo** | **mono** | **stereo** |  |
| N8 | 1 | 4 | 5 | 4 | 5 | 3 | 4 | 3 | 4 | stereo |
| N15 | 1 | 4 | 3 | 4 | 4 | 4 | 2 | 4 | 3 | stereo |
| N19 | 1 | 3 | 5 | 3 | 5 | 3 | 5 | 3 | 5 | stereo |
| N1 | 2 | 5 | 3 | 5 | 4 | 5 | 1 | 5 | 3 | mono |
| N2 | 2 | 4 | 3 | 4 | 5 | 4 | 3 | 4 | 4 | stereo |
| N20 | 2 | 4 | 5 | 5 | 5 | 5 | 1 | 3 | 3 | mono |
| N9 | 3 | 5 | 3 | 5 | 5 | 5 | 3 | 5 | 2 | mono |
| N18 | 3 | 5 | 3 | 5 | 5 | 5 | 2 | 3 | 3 | mono |
| N5 | 4 | 3 | 4 | 3 | 4 | 3 | 2 | 3 | 4 | n/a |
| N7 | 4 | 3 | 2 | 3 | 2 | 3 | 1 | 1 | 1 | mono |
| N4 | 5 | 5 | 5 | 5 | 2 | 5 | 1 | 5 | 3 | mono |
| N16 | 5 | 5 | 5 | 5 | 5 | 5 | 5 | 2 | 2 | mono |
| N22 | 5 | 4 | 1 | 4 | 1 | 4 | 1 | 4 | 1 | mono |
| N3 | 6 | 4 | 4 | 4 | 4 | 4 | 2 | 3 | 4 | stereo |
| N10 | 6 | 5 | 3 | 5 | 4 | 5 | 2 | 5 | 3 | mono |
| N17 | 6 | 5 | 3 | 5 | 2 | 5 | 5 | 3 | 3 | mono |
| N14 | 7 | 4 | 2 | 4 | 2 | 5 | 1 | 5 | 1 | mono |
| N12 | 8 | 5 | 3 | 5 | 2 | 5 | 2 | 5 | 2 | mono |
| N13 | 8 | 4 | 4 | 4 | 4 | 5 | 2 | 3 | 4 | n/a |
| N21 | 9 | 4 | 3 | 4 | 3 | 5 | 3 | 3 | 3 | mono |
| N6 | 12 | 5 | 4 | 5 | 5 | 5 | 3 | 5 | 5 | mono |
| N11 | 12 | 4 | 5 | 4 | 5 | 5 | 5 | 4 | 5 | stereo |
|  | **median** | 4 | 3 | 4 | 4 | 5 | 2 | 3.5 | 3 | n/a |
|  | **25th percentile** | 4 | 3 | 4 | 2 | 4 | 1 | 3 | 2 | n/a |
|  | **75th percentile** | 5 | 5 | 5 | 5 | 5 | 3.25 | 5 | 4 | n/a |

| **Supplemental Table 2. Results for questionnaire day 2: objective comparison of monoscopic vs. stereoscopic imaging method** | | | | | | | | | |
| --- | --- | --- | --- | --- | --- | --- | --- | --- | --- |
| **Participant** | **Group** | **T1 modality** | **T1 points** | **T2 modality** | **T2 points** | **A1 modality** | **A1 points** | **A2 modality** | **A2 points** |
| N1 | 1 | mono | 8 | stereo | 3 | mono | n/a | stereo | 7 |
| N2 | 2 | stereo | 4 | mono | 5 | stereo | 1 | mono | 6 |
| N3 | 1 | mono | 6 | stereo | 3 | mono | 2 | stereo | 6 |
| N4 | 2 | stereo | 5 | mono | 5 | stereo | 6 | mono | 7 |
| N5 | 1 | mono | 7 | stereo | 7 | mono | 6 | stereo | 5 |
| N6 | 2 | stereo | 5 | mono | 7 | stereo | 6 | mono | 6 |
| N7 | 2 | stereo | 3 | mono | 4 | stereo | 4 | mono | 5 |
| N8 | 1 | mono | 7 | stereo | 5 | mono | 5 | stereo | 6 |
| N9 | 2 | stereo | 5 | mono | 6 | stereo | 5 | mono | 7 |
| N10 | 2 | stereo | 6 | mono | 7 | stereo | n/a | mono | 6 |
| N11 | 1 | mono | 6 | stereo | 7 | mono | 6 | stereo | 6 |
| N12 | 2 | stereo | 6 | mono | 7 | stereo | n/a | mono | 5 |
| N13 | 1 | mono | 6 | stereo | 6 | mono | 5 | stereo | 6 |
| N14 | 1 | mono | 5 | stereo | 3 | mono | n/a | stereo | 7 |
| N15 | 2 | stereo | 7 | mono | 6 | stereo | 6 | mono | 6 |
| N16 | 1 | mono | 4 | stereo | 6 | mono | 5 | stereo | 7 |
| N17 | 1 | mono | 5 | stereo | 6 | mono | 5 | stereo | 4 |
| N18 | 2 | stereo | 7 | mono | 4 | stereo | n/a | mono | 4 |
| N19 | 1 | mono | 5 | stereo | 6 | mono | 3 | stereo | 5 |
| N20 | 1 | mono | 6 | stereo | 6 | mono | n/a | stereo | 6 |
| N21 | 2 | stereo | 7 | mono | 6 | stereo | 4 | mono | 7 |
| N22 | 2 | stereo | 0 | mono | 6 | stereo | n/a | mono | 6 |
| **median** | **Monoscopic** | | 6 |  | 6 |  | 5 |  | 6 |
| **25th percentile** |  |  | 5 |  | 5 |  | 3.5 |  | 6 |
| **75th percentile** |  |  | 6.75 |  | 6 |  | 5.75 |  | 7 |
| **median** | **Stereoscopic** | | 5 |  | 6 |  | 5 |  | 6 |
| **25th percentile** |  |  | 4 |  | 5.25 |  | 4 |  | 5 |
| **75th percentile** |  |  | 7 |  | 6.75 |  | 6 |  | 6 |
