# Supplementary material for: Does stereoscopic imaging improve the memorization of medical imaging by neurosurgeons? Experience of a single institution
Source: Neurosurg Rev. 2021 Sep 22;45(2):1371–81. doi: 10.1007/s10143-021-01623-0 (PMC8976776; doi:10.1007/s10143-021-01623-0)
Supplement: Supplementary file 2 — Supplementary file2 (PDF 80 KB) [file 10143_2021_1623_MOESM2_ESM.pdf]

# **Questionnaire** Comparison

*General rating - refers to all imaging viewed*

Year of training: ____ Initials: ____ stereo vision: _____

Please always rate the following statements on a scale from 1 (not at all) to 5 (completely) about stereoscopic or usual (monoscopic) imaging:

1. I can identify relevant pathological structures.

| | | | | | |
|---|---|---|---|---|---|
| stereoscopic: | 1 | 2 | 3 | 4 | 5 |
| monoscopic: | 1 | 2 | 3 | 4 | 5 |

2. I can identify relevant anatomical structures.

| | | | | | |
|---|---|---|---|---|---|
| stereoscopic: | 1 | 2 | 3 | 4 | 5 |
| monoscopic: | 1 | 2 | 3 | 4 | 5 |

3. I feel confident in using imaging.

| | | | | | |
|---|---|---|---|---|---|
| stereoscopic: | 1 | 2 | 3 | 4 | 5 |
| monoscopic: | 1 | 2 | 3 | 4 | 5 |

4. I have all the radiological information I need to perform an operation.

| | | | | | |
|---|---|---|---|---|---|
| stereoscopic: | 1 | 2 | 3 | 4 | 5 |
| monoscopic: | 1 | 2 | 3 | 4 | 5 |



Which of the two imaging modalities would you prefer:

[ ] monoscopic [ ] stereoscopic

What additional information would you need for surgery planning?

monoscopic:

______________________________________________

stereoscopic:

______________________________________________

What additional functions would you like for the respective image modality?

monoscopic:

______________________________________________

stereoscopic:

______________________________________________
