# Supplementary material for: Does stereoscopic imaging improve the memorization of medical imaging by neurosurgeons? Experience of a single institution
Source: Neurosurg Rev. 2021 Sep 22;45(2):1371–81. doi: 10.1007/s10143-021-01623-0 (PMC8976776; doi:10.1007/s10143-021-01623-0)
Supplement: Supplementary file 3 — Supplementary file3 (PDF 195 KB) [file 10143_2021_1623_MOESM3_ESM.pdf]

Questionnaire Tumor

2nd part: 2D / 3D

Year of training: ____   Initials: ____

1. Which side is the pathology on?
   right [ ]   left [ ]

2. What is the approximate size of the pathology?
   0-1cm [ ]   1-3cm [ ]   3-5cm [ ]   >5cm [ ]

3. Please roughly outline the level / location of the pathology:

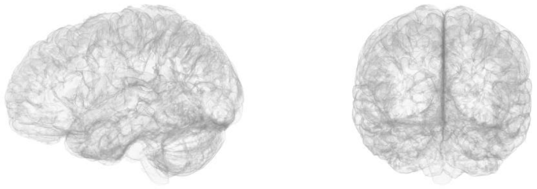

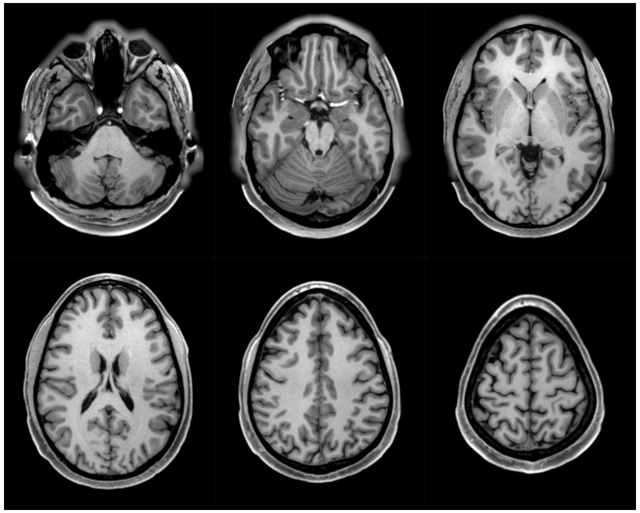

4. In which lobe is the tumor located?

   ____

5. Which functional area is most affected?

   ____

6. What type of tumor is it morphologically?

   ____

7. What is the relationship between the tumor and the tract shown?
   tumor touches trakt [ ]   tumor does not touch tract [ ]

8. Where is the tumor in relation to the tract shown?
   (Multiple selection possible)
   anterior [ ]   posterior [ ]   lateral [ ]   medial [ ]
