# Supplementary material for: Does stereoscopic imaging improve the memorization of medical imaging by neurosurgeons? Experience of a single institution
Source: Neurosurg Rev. 2021 Sep 22;45(2):1371–81. doi: 10.1007/s10143-021-01623-0 (PMC8976776; doi:10.1007/s10143-021-01623-0)
Supplement: Supplementary file 4 — Supplementary file4 (PDF 98 KB) [file 10143_2021_1623_MOESM4_ESM.pdf]

Questionnaire Aneurysm

2nd part: 2D / 3D

Year of training: ____ Initials: ____

1. Which side is the pathology on?
   right [ ] left [ ]

2. What is the approximate size of the pathology?
   0-1cm [ ] 1-3cm [ ] 3-5cm [ ] >5cm [ ]

3. Please roughly outline the level / location of the pathology:

A representative image of the circle of Willis

4. Which vessel is affected?

   ____

5. Can you provide more precise information about the location (e.g. segment)?

   ____

6. What is the morphology of the aneurysm?
   saccular [ ] fusiform [ ]

7. What is the relationship of the aneurysm to the adjacent vessels (e.g. bifurcation affected, outgoing vessels free)?

   ____
