# Supplementary material for: Does stereoscopic imaging improve the memorization of medical imaging by neurosurgeons? Experience of a single institution
Source: Neurosurg Rev. 2021 Sep 22;45(2):1371–81. doi: 10.1007/s10143-021-01623-0 (PMC8976776; doi:10.1007/s10143-021-01623-0)
Supplement: Supplementary file 5 — Supplementary file5 (DOCX 16 KB) [file 10143_2021_1623_MOESM5_ESM.docx]

**Supplemental Figure Legends**

Supplemental Fig. 1. Questionnaire day 1: subjective comparison of monoscopic versus stereoscopic imaging method.

Supplemental Fig. 2. Questionnaire day 2: objective comparison of monoscopic versus stereoscopic imaging method in tumor cases.

Supplemental Fig. 3. Questionnaire day 2: objective comparison of monoscopic versus stereoscopic imaging method in aneurysm cases.
